# Inflammatory Arthritis following Hepatitis B Vaccination in an Infant

**DOI:** 10.1155/2021/5598217

**Published:** 2021-04-07

**Authors:** Sana S. Rahimi, Barbara E. Ostrov, Maricarmen Lopez-Pena

**Affiliations:** ^1^Department of Primary Care Pediatrics, Nemours Children's Health System, Milford, DE, USA; ^2^Department of Pediatrics, Bernard and Millie Duker Children's Hospital at Albany Medical Center, Albany, NY, USA

## Abstract

Inflammatory arthritis in children may be idiopathic in nature or may be due to or follow infections. Rare reports identify inflammatory arthritis temporally related to vaccination in children. Herein, we describe the first reported case of an infant who developed inflammatory arthritis following hepatitis B vaccination. A 10-day-old female presented for evaluation of decreased movement of the right lower extremity and right knee swelling. Of note, the patient received a hepatitis B vaccine in her right thigh at birth. A workup found the patient to have a negative ANA but the presence of HLA B27. Findings resolved using ibuprofen. A literature review identified reports of what has been termed “reactive arthritis” in adult patients following the hepatitis B vaccine, frequently in association with HLA B27. No prior pediatric cases have been published. Healthcare providers must be aware of the rare development of postvaccination inflammatory arthritis.

## 1. Introduction

Childhood inflammatory arthritis can have several causes. Among those causes include chronic conditions such as juvenile idiopathic arthritis (JIA), which presents with inflammation lasting for at least 6 weeks prior to a child's 16^th^ birthday. Acute inflammatory arthritis due to infections, medications, and vaccinations is also noted in the literature. For example, arthritis is well described with Lyme disease, gonorrhea, salmonella, and several viruses. The rubella vaccine, which is a live attenuated vaccine, can also cause an inflammatory arthritis [1]. Although there is no live hepatitis B virus in the vaccine directed against this pathogen, the literature does describe inflammatory arthritis, termed “reactive arthritis,” in adult patients after hepatitis B vaccination. This finding is reported within higher frequency in patients carrying the human leukocyte antigen HLA B27. In the report herein, we present the first case in the literature of an infant who developed inflammatory arthritis soon after hepatitis B vaccination and review the literature on this association. Our objective in reporting this case is to ensure that healthcare providers are aware of this potential reaction in infants and children and particularly in those who carry arthritogenic HLA haplotypes.

## 2. Case

**Figure 1:**
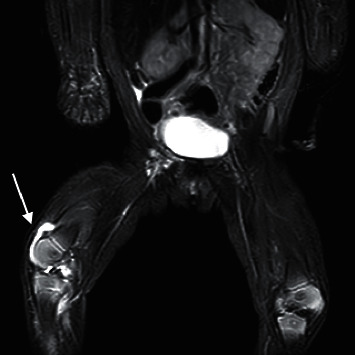
MRI of the patient's right knee showing small to moderate right knee effusion compared to a normal left knee. There is no myositis or soft tissue fluid changes.

A previously healthy Caucasian female presented to her pediatrician with a decreased movement of the right lower extremity. The patient was born at 36 weeks for maternal preeclampsia. She received the hepatitis B vaccine in the right thigh prior to newborn nursery discharge. The patient kept her right knee and hip in a flexed position since discharge. Her primary care provider initially believed refusal to move the right lower extremity was due to right hip flexor tightness. A hip ultrasound was normal. The infant then presented to the Children's Hospital at day 10 of life and was noted to have right knee swelling. Her initial set of labs were significant for white cell count 11,000 cells/mm3, hemoglobin 14.1 g/dl, platelets 463,000, ESR 18 mm/hr (normal < 15 mm/hr), and C-reactive protein 4.1 mg/L (normal < 8 mg/L). A knee MRI revealed a right knee effusion (Figure 1) and no myositis or soft tissue fluid changes. Arthrocentesis was performed; culture was negative after 5 days.

The patient remained afebrile throughout the hospital course and never received antibiotics. She was treated with ibuprofen 10 mg/kg/dose three times a day. Additional testing included negative Lyme titer, negative parvovirus IgG and IgM, normal ferritin, normal total IgA, IgG, and IgM levels, and negative ANA; however, she was found to have a positive HLA B27. The patient was discharged after 2 days following the negative evaluation and some increased knee range of motion. On follow-up, she was noted to have improvement of her right knee swelling and increased mobility of the right lower extremity. She was prescribed physical therapy.

The mother reported that she had regular prenatal care. The only perinatal complication was preeclampsia for which she was induced at 36-week gestation. The infant passed her hearing and critical congenital heart disease screen. New York Newborn Metabolic screen was completely negative.

Maternal history was significant for ADHD and bipolar depression, for which she was prescribed sertraline during pregnancy. Mother had negative tests for parvovirus, Lyme, SS-A/RO, and SS-B/LA antibodies and ANA. There is no known family history of autoimmune diseases such as psoriasis, rheumatoid arthritis, or inflammatory bowel disease (IBD).

After 3 weeks of ibuprofen and physical therapy, the patient had significantly improved knee swelling and movement of the lower extremity; however, there was residual mild inflammation and stiffness of the knee. Since the patient had clinically improved, the family stopped ibuprofen despite recommendation to continue therapy.

The family moved out of state shortly after the 2-month visit. Discussion with the patient's mother revealed a normal interval history, meeting all milestones and with an age-appropriate gait at age 13 months. A pediatric rheumatologist at the local children's hospital evaluated the patient, and no joint findings were identified. They recommended monitoring for recurrence of arthritis as she is positive for HLA B27. The mother also reported that the infant received all remaining vaccines, including the hepatitis B vaccine series, without any negative consequences.

This case presents a very young infant who developed an inflammatory arthritis believed to be secondary to the initial hepatitis B vaccine.

## 3. Discussion

We present the first case in the literature of an infant who developed inflammatory arthritis soon after hepatitis B vaccination, similar to reports in the literature of adults with postvaccine articular complications [2–6]. We considered the possibility that this child had a very early presentation of JIA in the presence of HLA B27 positivity. However, JIA typically would not demonstrate such significant improvement within 3 weeks using just NSAIDs. Additionally, there was no known family history of psoriasis or IBD, making other forms of JIA typically associated with HLA B27 less likely. Autoinflammatory diseases also need to be considered; however, our patient had no rash or fevers and no such features were reported at the 13-month follow-up discussion.

### 3.1. Inflammatory Arthritis after Hepatitis B Vaccination

Inflammatory arthritis in children can have several causes, among them including JIA, as reviewed above. Other common causes include infections such as Lyme disease, gonorrhea, salmonella, and several viruses. Other triggers for inflammatory arthritis can include medications and vaccinations. The rubella vaccine, which is a live attenuated vaccine, can cause transient arthritis [1] The hepatitis B vaccine has also been reported to cause an inflammatory arthritis in adults, termed “reactive arthritis” in the literature [2–6]. Reactive arthritis is classically nonseptic inflammatory arthritis following an infection. The infection usually occurs about 1–4 weeks prior to the onset of arthritic or enthesitic symptoms. The subsequent inflammatory symptoms may persist for weeks to months and are often found in patients who carry HLA B27 [6]. The specific pathogenesis of reactive postinfectious and noninfectious inflammatory arthritis is still not clear. Several hypotheses suggest that HLA B27 produces a predilection to developing reactive arthritis due to misfolding of the HLA B27 molecule [7]. Another hypothesis suggests the location of expression of HLA B27 increases the host's inflammatory response leading to postinfectious arthritis [7]. Molecular mimicry from adjuvants used to stimulate the immune response during vaccination may also play a role in developing autoimmunity as suggested in patients with multiple sclerosis or lupus, particularly in those who carry certain HLA haplotypes [4]. We hypothesize there may be similar processes leading to the development of postvaccination inflammatory arthritis.

Soon after the hepatitis B vaccine first became available in 1982 [8, 9], several cases of adults developing arthritis symptoms were reported after the administration of this vaccine; however, there do not appear to be any reported cases in infants or children. The adult literature has termed this post-hepatitis B vaccine-related inflammatory arthropathy as “reactive arthritis” [2–6], even though this vaccine is not developed with attenuated whole virus and there is no actual hepatitis B virus in the vaccine. Several published studies showed that those who had underlying HLA alleles such as B27, DR1, DR4, or other class II haplotypes were more likely to develop inflammatory or “reactive” arthritis [2–5]. No pediatric reports were identified. The presence of HLA B27 was also associated with more severe arthritis [2]. A French 1999 study of 22 patients suggested that the hepatitis B vaccine may be the trigger to “unmask” rheumatic diseases. These patients developed rheumatoid arthritis, reactive arthritis, erythema nodosum with oligoarthritis, lupus, polyarthralgia-myalgia, vasculitis, and sicca syndrome [4].

In children with preexisting diagnoses of JIA, studies demonstrate the safety and efficacy of the hepatitis B vaccine [10, 11]. These studies showed that the hepatitis B vaccine series was safe and none of the children had a JIA flare temporally related to the vaccine series. There are no reports suggesting “unmasking” of JIA or other inflammatory arthritis in children following hepatitis B vaccination.

### 3.2. Very Early-Onset JIA

It is possible that our patient had very early-onset JIA with an oligoarticular presentation. The different JIA subtypes include oligoarticular arthritis, psoriatic arthritis, and enthesitis-related arthritis (ERA). Our patient's presentation would be most consistent with oligoarticular JIA, which is most frequently seen in young Caucasian females, although typically the ANA is positive rather than HLA B27. Oligoarticular JIA most frequently presents between 2 and 5 years of age. A review of the literature was completed to find the youngest documented cases of JIA. Identified studies report the youngest patients are 6 months of age typically. Some young patients are positive for HLA B27 although usually associated with the ERA subtype and not typically oligoarticular JIA as would be the diagnostic consideration in our patient. There are no reports of infants as young as our patient with JIA in the literature [12–14].

## 4. Conclusion

This is the first reported case of an infant developing an inflammatory arthritis after receiving the hepatitis B vaccine. The patient most likely had vaccine-related inflammatory arthritis rather than JIA given the significant improvement after a short course of NSAIDs and lack of recurrence which would be expected if the arthropathy was due to early-onset JIA. There are reports of increased vulnerability to developing such an inflammatory or reactive arthritis in adult patients who are HLA B27 positive as in our patient [2, 6]. In addition, the literature suggests that the hepatitis B vaccination can unmask autoimmune processes in adult patients although this has not been reported in children. Healthcare providers must be aware of the rare occurrence of post-hepatitis B vaccine inflammatory “reactive” arthritis when caring for young children, especially those known to carry arthritis risk HLA alleles.
